# *Trypanosoma cruzi* load in synanthropic rodents from rural areas in Chile

**DOI:** 10.1186/s13071-018-2771-2

**Published:** 2018-03-12

**Authors:** Esteban Yefi-Quinteros, Catalina Muñoz-San Martín, Antonella Bacigalupo, Juana P. Correa, Pedro E. Cattan

**Affiliations:** 1 Laboratorio de Ecología, Departamento de Ciencias Biológicas Animales, Facultad de Ciencias Veterinarias y Pecuarias, Universidad de Chile, Santiago, Chile; 2 Laboratorio de Ecología Evolutiva, Departamento de Ciencias Ecológicas, Facultad de Ciencias, Universidad de Chile, Santiago, Chile

**Keywords:** Chagas disease, Quantitative real-time PCR, Parasitemia, Host-parasite relations, *Rattus rattus*

## Abstract

**Background:**

*Trypanosoma cruzi* is the agent of Chagas disease, a major public health problem in Latin America. Many wild and domestic animals are naturally infected with *T. cruzi*; rodents are one of the groups which have been consistently detected infected in different countries. The aim of this work was to characterize blood *T. cruzi* load in naturally infected rodents from a Chagas disease endemic region in Chile.

**Methods:**

Baited traps were set in domestic and peridomestic areas of rural dwellings. The rodents were anesthetized and blood sampled; DNA was extracted and the parasite load was quantified by *T. cruzi* satellite DNA real-time PCR assays.

**Results:**

Seventy-one rodents of four species, *Rattus rattus*, *Mus musculus*, *Phyllotis darwini* and *Octodon degus*, were captured; *R. rattus* was the most abundant species. Fifty-nine samples (83.1%) were *T. cruzi*-positive and the median value of the parasite load was 2.99 parasite equivalents (par-eq)/ml. The comparison of frequency of infection or parasite load by species showed no differences. However, one *R. rattus* presented very elevated parasitemia (1644 par-eq/ml).

**Conclusions:**

The overall levels of parasitemia were similar to those found in humans in Chile. The high infection levels in exotic and endemic rodents very near to rural settlements increases their relevance as *T. cruzi* hosts.

**Electronic supplementary material:**

The online version of this article (10.1186/s13071-018-2771-2) contains supplementary material, which is available to authorized users.

## Background

The protozoan parasite *Trypanosoma cruzi* is the causative agent of Chagas disease, a complex zoonosis transmitted by about 150 hematophagous triatomine species and supported by more than 100 species of mammals, from the southern United States to Argentina and Chile [1]. This neglected vector-borne disease is one of the most important parasitic infections in Latin America [2].

North-Central Chile (17°50'S to 34°36'S) is considered a Chagas disease endemic zone, where the protozoan *T. cruzi* has four vector species [3, 4] and infects several endemic and exotic mammals [5].

Rodents act as hosts of the parasite and play a role in the transmission of *T. cruzi* by connecting the sylvatic, peridomiciliary and domiciliary cycles [6], thus transporting parasites to human dwellings where they are transmitted to humans and domestic animals by triatomines [7]. The frequency of infection by *T. cruzi* in exotic and endemic rodents in Chile has been estimated previously by detecting mitochondrial kinetoplastic DNA of *T. cruzi* with conventional PCR assays, reaching levels of infection about 25–41% in *Phyllotis darwini* [8, 9], 13–70% in *Octodon degus* and 27% in *Rattus rattus* [5, 8] in sylvatic areas. Their relevance as hosts of *T. cruzi* in Chile has not been established previously in the peridomestic and domestic environment. In this work we evaluated the parasitic load in rodents from an endemic area for Chagas disease, discussing their role as hosts of *Trypanosoma cruzi*.

## Methods

### Collection area

**Fig. 1:**
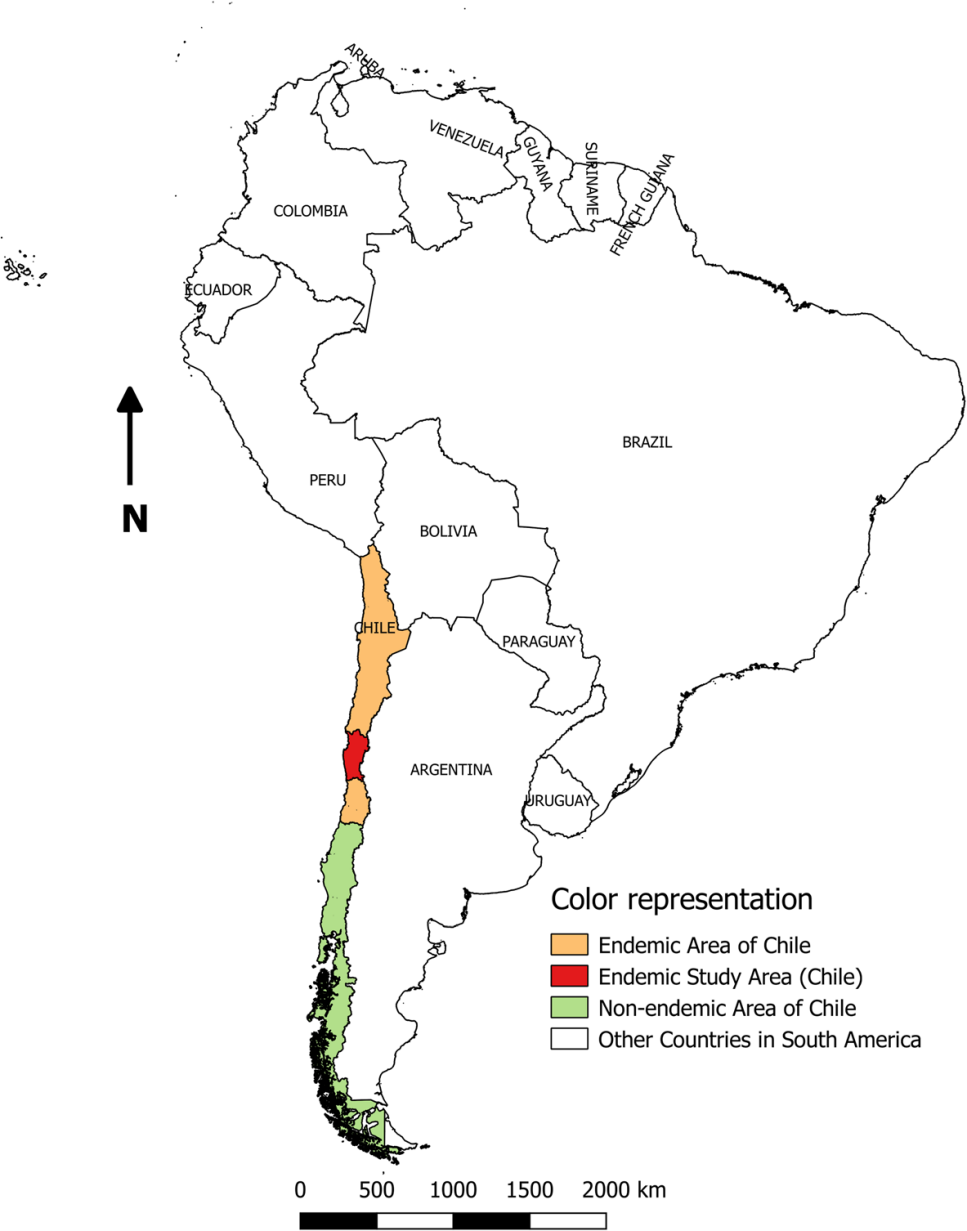
Location of the study region within the endemic area for Chagas disease in Chile, South America

**Fig. 2:**
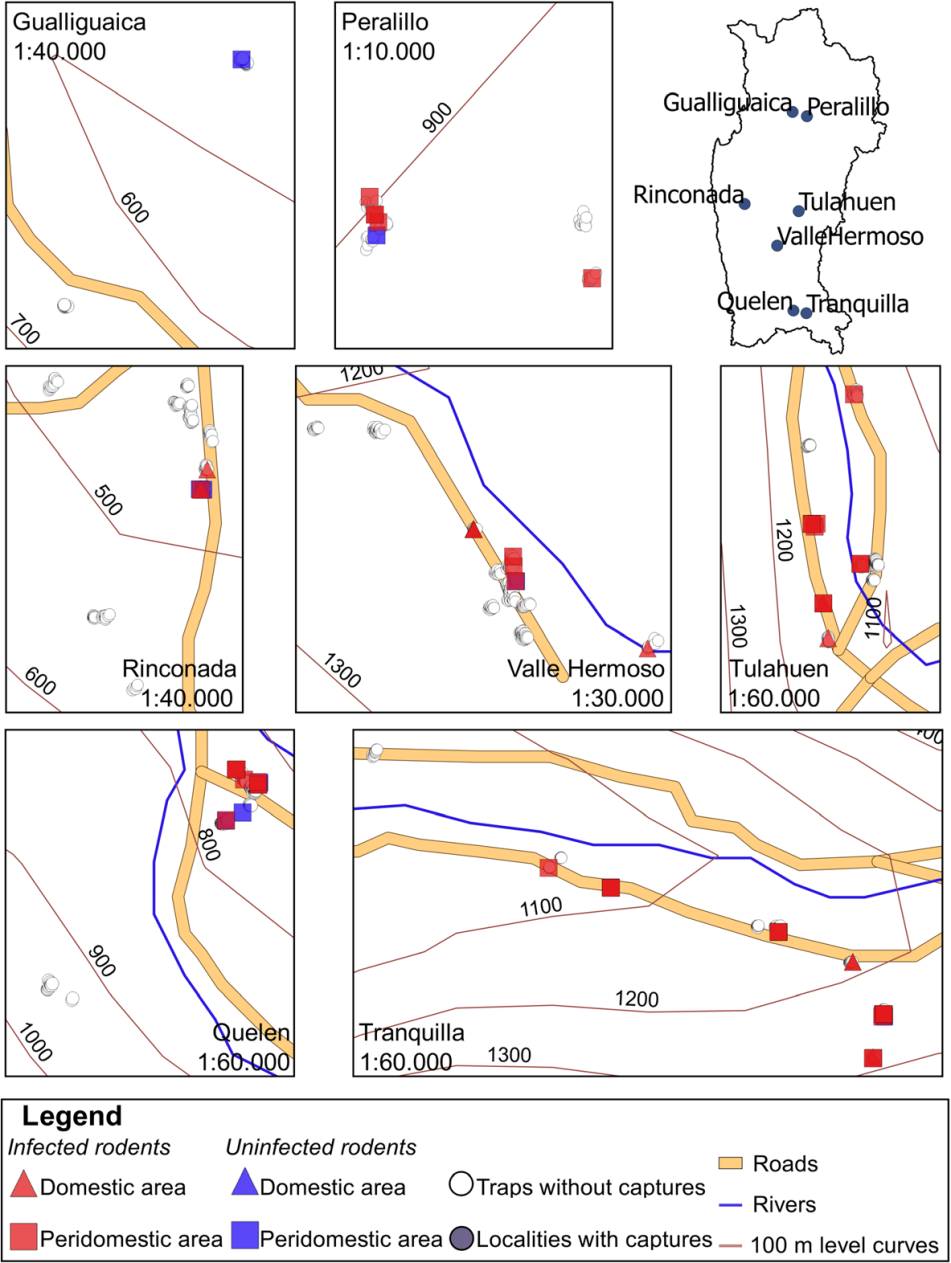
Location of the rodents by trapping area and infection status in each studied locality, within the study region

Rodents were collected from Coquimbo Region, a Chagas disease endemic zone located in the North-Central zone of Chile (Fig. 1), which is characterized by an arid Mediterranean climate. The rain is concentrated in winter, between May and August, but it has a marked water deficit during most of the year. Its geography is characterized by a series of mountain ranges and transverse valleys with rivers. The north-facing slopes are particularly exposed, and have low vegetation cover; on the south-facing exposure slopes thick grasses and shrubs are frequent. The valleys, on the other hand, have fertile soils [10]. The studied localities have an altitude range between 300–1800 m above sea level (Fig. 2).

### Trapping procedures

Domestic (inside dwellings) and peridomestic (areas around the house with permanent or transitory structures within the property limits) areas of rural dwellings were prospected using Tomahawk™ (WI, USA), Sherman™ (FL, USA) and Rodentrap™ traps (Santiago, Chile) baited with rolled oats, and provided with cotton bedding. Trapping was conducted from December 2014 through February 2015 and in January 2016, corresponding to the austral summer. The total number of traps set in each dwelling, including domestic or peridomestic areas ranged between 12 and 16. They were placed according to the availability of sites with shadow outside the house, and the permission of the owners to place traps inside; within the dwelling, the maximum traps set were 6. The traps were activated for 3 nights with a total capture effort of 3315 trap-nights. The number of traps set in domestic or peridomestic areas per locality is detailed in Additional file 1: Table S1. Prospected peridomestic areas were sites near each house, adjacent, within or underneath firewood, shrubs, animal corrals, chicken coops, rock piles, construction materials, warehouses and food or waste accumulations. Inside dwellings, traps were set in bedrooms, kitchens, bathrooms, dining rooms and attics.

### Biological samples

Peripheral blood samples of 71 rodents were incorporated in the study. They were examined and anesthetized according to species: the exotic rodents (*Rattus rattus* and *Mus musculus*) were anesthetized with isoflurane, and endemic rodents (*Phyllotis darwini* and *Octodon degus*), with isoflurane induction, and intramuscular Ketamine-Xylazine anesthesia (40–85 mg/kg + 5–21 mg/kg). All the specimens were weighed, measured (total body length and tail length) and their sex was determined. Blood samples were obtained by puncture of the saphenous vein (*O. degus*), masseteric vein (*P. darwini*) or cardiac puncture (*R. rattus* and *M. musculus*). A minimum of 0.2 ml of blood sample was obtained from each rodent and immediately preserved with an equal volume of 6 mol/l guanidine-HCl-0.2 mol/l EDTA solution (GEB samples). After sampling, endemic rodents were ear-tagged and released at the point of capture or in the vicinity, if they were captured indoors, and exotic rodents were euthanized by anesthesia overdose. The samples were stored in the laboratory at 4 °C until DNA extraction.

### DNA extraction

DNA extraction from 200 μl of GEB samples was performed using the UltraCleanTM BloodSpin™ Kit (MO BIO, CA, USA) according to the manufacturer’s instructions, with 100 μl of solution B5 in the elution step. Total DNA concentration from biological samples was quantified to assess the amount and integrity of genomic DNA [11, 12] using Qubit® dsDNA HS Assay Kit (Life Technologies, OR, USA) according to the manufacturer’s instructions. The eluted samples were stored at -20 °C until qPCR assays.

### *Trypanosoma cruzi* satellite DNA real-time PCR assays

The parasite load was quantified in each sample by qPCR assays using *T. cruzi* nuclear satellite DNA primers Cruzi 1 and Cruzi 2 [13]. Assays were performed in a Rotor-Gene® Q (Qiagen, CA, USA), with a final volume of 20 μl containing 2 μl DNA template, 5× HOT FIREPol® EvaGreen® qPCR Mix Plus (Solis BioDyne, Tartu, Estonia), 0.3 μM of each primer and nuclease free water. The cycling conditions were: a pre-incubation for 15 min at 95 °C, followed by 40 cycles at 95 °C for 15 s, 65 °C for 20 s and 72 °C for 20 s. After all amplification cycles, a melting curve was run. Each sample was tested in duplicate.

Considering that one parasite cell harbors approximately 200 fg of DNA [14, 15], the standard curve for absolute quantification was performed with a 10-fold serial dilution of the DNA extracted from a *T. cruzi* free *R. rattus* GEB sample spiked with 10^6^ parasite equivalents/ml (par-eq/ml) [16], so the standard was submitted to an equivalent loss of genomic DNA due to the extraction process as the samples. Given the variability in the number of copies of the nuclear satellite DNA previously described [14, 16, 17], the curve was made with equal quantities (20 ng each/200 μl blood) of the clonal reference strains Dm28c (TcId) and Y (TcII) to reduce the differences in the detection limits; one strain from the group with the highest number of copies (TcII) and one strain of the group with the lowest number of copies (TcI) were mixed [18–20].

### Statistical methods

The body mass index (BMI) of each rodent was calculated as BMI = mass/(total length - tail length)^2^ [5], and was tested for normality and homogeneity of variance. We analysed the frequency of infection by species and by sex, with the Fisher’s exact test (Statacorp. 2005. Stata Statistical Software: Release 9.1. College Station, TX, USA: Statacorp LLC.), and we tested if the BMI was different according to infection status with the unpaired Student's t-test. The parasite load was evaluated by species using the Kruskal-Wallis H-test, and by sex, with the Mann Whitney U-test. The BMI and *T. cruzi* load correlation was evaluated by the Spearman’s test (GraphPad Prism version 7.00 for Windows, GraphPad Software, La Jolla CA, USA, https://www.graphpad.com/). All the tests were performed with a significance level of α = 0.05.

## Results

### Trapping

Seventy-one rodents were captured in domestic or peridomestic areas: 55 *R. rattus*, six *M. musculus*, six *P. darwini* and four *O. degus* (Table 1). The location of the captured rodents and their infection status are shown in Fig. 2. In two localities there were no rodents captured (Cochiguaz and Matancilla, localities not shown in Fig. 2). The captured rodents were 28 females and 43 males, with a mean BMI of 0.29 g/cm^2^ (SD ± 0.08).

**Table 1:** Description of the rodents captured in rural dwellings of Coquimbo Region, Chile.

Species	Sex		Trapping area *n* (%)		Infection status *n* (%)		BMI	Median parasite load (range)
	F	M	Domestic	Peridomestic	Infected	Uninfected	Mean ± SD (g/cm^2^)	(par-eq/ml)
*Rattus rattus*	22	33	10 (18.2)	45 (91.8)	46 (83.6)	9 (16.4)	0.29 ± 0.07	3.27 (0.07–1644.34)
*Mus musculus*	2	4	0 (0.0)	6 (100)	5 (83.3)	1 (16.4)	0.19 ± 0.02	2.45 (1.67–8.52]
*Phyllotis darwini*	2	4	2 (33.3)	4 (66.7)	6 (100)	0 (0)	0.30 ± 0.05	3.37 (2.21–6.89)
*Octodon degus*	2	2	1 (25.0)	3 (75.0)	2 (50.0)	2 (50.0)	0.40 ± 0.04	3.40 (1.64–5.15)
Total	28	43	13 (18.3)	58 (81.7)	59 (83.1)	12 (16.9)	0.29 ± 0.08	3.23 (0.07–1644.34)

*Abbreviations*: *F* female, *M* male

### DNA concentration

Genomic DNA was detected in all extracted samples, and their concentration values ranged between 0.11–28.2 ng/μl (mean 3.16, SD ± 4.84).

### *Trypanosoma cruzi* satellite DNA quantitative real-time PCR assays

All captured rodents (*n* = 71) were evaluated using qPCR assays. Overall 59 rodent samples (83.1% of captures) were positive to *T. cruzi*: 46 *R. rattus* (83.6% of the captures of this species), 5 *M. musculus* (83.3%), 6 *P. darwini* (100%) and 2 *O. degus* (50%) (Table 1). According to sex, 25 (89.3%) females and 34 (79.1%) males were infected. The status of *T. cruzi* infection did not show significant differences by species (Fisher’s exact test: *P* = 0.225) or by sex (Fisher's exact test: *P* = 0.341). Normality (Shapiro-Wilk: infected *W* = 0.98, *P* = 0.377; uninfected *W* = 0.98, *P* = 0.955) and homogeneity of variance (Levene test: *w0* = 1.97; *df*_(1, 63)_; *P* = 0.165) of the BMI according to infection status were not rejected. BMI of infected (mean 0.29 g/cm^2^, SD ± 0.08) and uninfected (mean 0.32 g/cm^2^, SD ± 0.10) did not show significant differences (unpaired t-test: *t*_(63)_ = 1.41, *P* = 0.121).

**Fig. 3:**
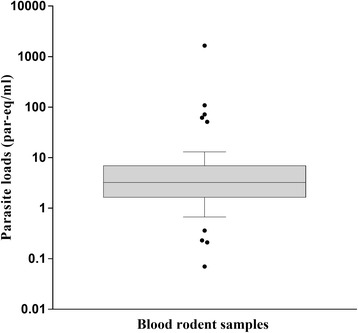
*Trypanosoma cruzi* loads in rodent blood samples. Parasite loads are represented in logaritmic scale as parasite equivalents/ml. The line inside the box plot represents the median, and the box extends from lower to upper quartiles. Whiskers indicate the 90th and 10th percentiles and dots represent the outliers

In the quantification, the threshold cycle (Ct) values ranged between 25.68–36.45 (mean 30.76, SD ± 1.93). Parasite loads in the 59 positive samples were calculated using the standard curve of GEB sample spiked with a mixture of strains (efficiency = 0.98, *R*^2^ = 0.993), and fluctuated between < 1 and 109 par-eq/ml with a median value of 2.99 par-eq/ml (Fig. 3). An interesting finding was one outlier (*R. rattus*) with a high parasite load (1644 par-eq/ml). The parasite loads did not show significant differences when evaluating by species (Kruskal-Wallis H-test: *χ*^2^ = 0.208, *df* = 3, *P* = 0.976) or by sex (Mann-Whitney U-test: *U* = 417.5, *Z* = -0.12, *P* = 0.912). No correlation between the body mass index and the parasite load was obtained (Spearman's *r* = -0.27, *P* = 0.059). Results by individual are included in Additional file 2: Table S2.

## Discussion

The main way of transmission of *T. cruzi* in rodents from Chile is supposed to be vectorial, and *Mepraia spinolai* is the most important vector in the wild, reaching infection levels up to 76% [21]. Hence, this is likely to be the main way *T. cruzi*-infected rodents became infected. Also, the oral route is probably a frequent form of circulation of the parasite in the wild cycle by carnivorous, insectivorous and licking behavior [22]. The protozoan infection of rodents did not differ between sexes, which agrees with a previous report [5]; however, female rodents of the most abundant species (*R. rattus*) might have a greater effect on the long-term transmission of *T. cruzi* by means of their high reproductive ratio, conjugated with the possible propagation of the agent to their offspring, as was described for *Rattus norvegicus* in experimental infections [23], maintaining the parasite in time.

Rodents are frequently found in triatomines’ blood meals [24, 25]. If the rodent species sampled in our study are a food source for triatomines, their high infection levels with *T. cruzi* may be indicating that wild or peridomestic triatomines are getting these parasites through their blood meals, and in this way they are favoring the transmission of *T. cruzi* in the wild and peridomestic cycles. The existence of animal food sources in the peridomicile or even inside dwellings has the possible consequence of permitting the establishment and maintenance of vector colonies in these areas. Particularly, this study shows that *R. rattus* is a relevant host in domestic and peridomestic cycles in Chagas disease endemic zones, because of its high frequency of infection and its higher population abundances [26]. However, we also found the presence of the endemic species *O. degus* and *P. darwini*, usually classified as wild, being very close to dwellings, breaking the theoretical limits of the wild and domestic cycles.

The quantification of *T. cruzi* in rodents’ blood revealed that their levels of parasitemia were similar to those found in *O. degus* from sylvatic areas (median value: 6.2 par-eq/ml) [27] and humans. The median value in Chilean human samples (6.42 par-eq/ml) [19], and the median values found in Colombian and Argentinian human samples (2.3 and 1.9 par-eq/ml, respectively) [16] is not far from that detected in this study, of 2.99 par-eq/ml. Interestingly, there were four rodents with parasite loads ranging between 51–109 par-eq/ml, which are similar to the loads reported for chronic Chagas cardiomyopathy patients [16]. The outlier load (1644 par-eq/ml) approaches the loads found in HIV/*T. cruzi* co-infection patients with Chagas disease reactivation (median value: 1428 parasites/ml) [28] and in acute congenital Chagas in newborns (> 1000 parasites/ml) [29]. *Trypanosoma cruzi* load in rodents’ blood is not related to species, sex or BMI, so it is probable that the immune response regulates the parasite load in these species [30]. It can also be related to the parasite load inoculated when the rodents got infected [31], which was not evaluated here.

We showed that rodents are relevant hosts for *T. cruzi* in domestic and peridomestic locations of Chagas disease endemic areas, without discarding that other mammals may be playing a role in the parasite’s cycle. We propose that the maintenance of *T. cruzi* in North-Central Chile is ensured by the abundance of different native and exotic rodent species and their high levels of infection [8, 26, 32]. In fact, rodents may be used as sentinels, as their chronic infections [5] allow detection of exposure to the pathogen in an extended temporal manner, reflecting the risk of infection in local areas. Furthermore, the simple way that they can be captured and sampled facilitates their use to detect *T. cruzi*. In particular, exotic species such as *M. musculus* and *R. rattus* relate closely with human activities [33], and those species could be removed from these areas by trapping procedures, given that they are considered plagues because of their risk of transmitting several pathogens [34]. The removal of rodents from within and around dwellings in other settings has reduced the risk of having vector colonies, thus diminishing the risk of human vectorial transmission of *T. cruzi* [35]. In this context, rodent control of exotic species in areas occupied by people is highly recommended.

## Conclusions

Our study confirmed that *T. cruzi* circulates very near to rural human population in the studied area, with rodents acting as relevant hosts for the parasite in domestic and peridomestic locations. In fact, 83.1% of rodents were infected with parasite loads similar to those in human cases. Rodent control of exotic species should be encouraged, along with vector control, given that triatomines will probably be infected as well.

## Additional files

Additional file 1: **Table S1.** Number of traps set per locality, by trapping area. (DOCX 16 kb)

Additional file 2: **Table S2.** Results per individual. (XLSX 14 kb)

## Abbreviations

DNA: Deoxyribonucleic acid
PCR: Polymerase chain reaction
par-eq/ml: Parasite equivalents per millilitre
EDTA: Ethylenediaminetetraacetic acid
GEB: Guanidine hydrochloride EDTA blood
dsDNA: Double-stranded DNA
qPCR: Quantitative real-time PCR
BMI: Body mass index
SD: Standard deviation
Ct: Cycle threshold
HIV: Human immunodeficiency virus
