# Supplementary material for: Trypanosoma cruzi load in synanthropic rodents from rural areas in Chile
Source: Parasit Vectors. 2018 Mar 12;11:171. doi: 10.1186/s13071-018-2771-2 (PMC5848452; doi:10.1186/s13071-018-2771-2)
Supplement: Supplementary file 1 — Table S1. Number of traps set per locality, by trapping area. (DOCX 16 kb) [file 13071_2018_2771_MOESM1_ESM.docx]

| Localities | Trapping Area | | Total |
| --- | --- | --- | --- |
|  | **Domestic** | **Peridomestic** |  |
| Gualliguaica | 30 | 31 | 61 |
| Peralillo | 30 | 26 | 56 |
| Cochiguaz | 30 | 27 | 57 |
| Tulahuen | 78 | 115 | 193 |
| Valle Hermoso | 96 | 101 | 197 |
| La Rinconada | 78 | 80 | 158 |
| Matancilla | 78 | 80 | 158 |
| Tranquilla | 60 | 63 | 123 |
| Quelen | 42 | 60 | 102 |

**Additional file 1: Table S1.** Number of traps set per locality, by trapping area.
